# City 5.0: Citizen involvement in the design of future cities

**DOI:** 10.1007/s12525-023-00621-y

**Published:** 2023-04-27

**Authors:** Jörg Becker, Friedrich Chasin, Michael Rosemann, Daniel Beverungen, Jennifer Priefer, Jan vom Brocke, Martin Matzner, Adela del Rio Ortega, Manuel Resinas, Flavia Santoro, Minseok Song, Kangah Park, Claudio Di Ciccio

**Affiliations:** 1 Information Systems, University of Muenster (WWU), Leonardo-Campus 3, 48149 Muenster, Germany; 2 Information Systems and Systems Engineering, University of Cologne, Pohligstr. 1, 50969 Cologne, Germany; 3 Information Systems, Queensland University of Technology (QUT), 2 George St, Brisbane City, QLD 4000 Australia; 4 Business Information Systems, Paderborn University, Warburger Str. 100, 33098 Paderborn, Germany; 5 Information Systems and Business Process Management, University of Liechtenstein, Fuerst-Franz-Josef-Strasse, 9490 Vaduz, Liechtenstein; 6 Digital Industrial Service Systems, University of Erlangen–Nuremberg (FAU), Schlossplatz 4, 91054 Erlangen, Germany; 7 Computer Languages and Systems, University of Seville, C. San Fernando, 4, 41004 Sevilla, Spain; 8 Applied Informatics, Federal University of the State of Rio de Janeiro, R. Sao Francisco Xavier, 524 - Maracana, Rio de Janeiro, RJ 20550-013 Brazil; 9 Industrial and Management Engineering, Pohang University of Science and Technology (POSTECH), 77 Cheongam-Ro, Nam-Gu, Gyeongbuk 37673 Pohang, South Korea; 10 Computer Science, Sapienza University of Rome, Piazzale Aldo Moro, 5, 00185 Roma, Italy

**Keywords:** Citizen involvement, Smart city, City 5.0, Restrictions, Future cities, O18

## Abstract

A citizen-centric view is key to channeling technological affordances into the development of future cities in which improvements are made with the quality of citizens’ life in mind. This paper proposes City 5.0 as a new citizen-centric design paradigm for future cities, in which cities can be seen as markets connecting service providers with citizens as consumers. City 5.0 is dedicated to eliminating restrictions that citizens face when utilizing city services. Our design paradigm focuses on smart consumption and extends the technology-centric concept of smart city with a stronger view on citizens’ roadblocks to service usage. Through a series of design workshops, we conceptualized the City 5.0 paradigm and formalized it in a semi-formal model. The applicability of the model is demonstrated using the case of a telemedical service offered by a Spanish public healthcare service provider. The usefulness of the model is validated by qualitative interviews with public organizations involved in the development of technology-based city solutions. Our contribution lies in the advancement of citizen-centric analysis and the development of city solutions for both academic and professional communities.

## Citizen involvement through the City 5.0 paradigm

“To look at citizens’ restrictions that are not created by the city itself but are present because the citizen is an individual (with all that comes with it) is a fascinating approach to me.”

[Chief Digital Officer, City of Paderborn, P:5^1^]

When public administrations use technology to create services intended to benefit citizens, success is not assured. Such initiatives can fail to improve the well-being of citizens or may even create entirely new technological barriers. A citizen does not benefit from a technological solution per se. The benefit comes rather from improving the citizen’s liveability within the environment. This is best illustrated by a private–public partnership initiative in Queensland, Australia. Using streaming and broadcasting technologies, a collaboration of local government and private organizations was able to increase cultural access by enabling citizens across the vast state of Queensland to view theater performances in Brisbane from 16 locations up to 1000 km away (Queensland Cabinet and Ministerial Directory (CMD), 2019).

This solution emerged from the desire to overcome spatial constraints faced by citizens of one of the most urbanized countries in the world. Queensland is five times the size of Japan and seven times that of the UK. As a result, Queensland citizens have limited access to events in the capital city of Brisbane. In addition, many citizens are unable to afford high-quality cultural events, which therefore tend to cluster in economically strong cities. To overcome these limitations, the Queensland Performance and Arts Centre (QPAC), with the support of the Queensland government and local private organizations, provides regular simulcasts, bringing prestigious performances (e.g., international ballet performances) held in Brisbane to the Queensland region. The service brought cultural events to citizen groups beyond those who could afford a ticket to the actual performance.

Providing urban residents with the means and resources for a “good life” and offering adequate options to citizens in remote areas is a major concern for improving the human habitat (Portmann et al., 2018). Access to a comprehensive range of high-quality public goods and services contributes to citizens’ quality of life along various dimensions of quality of life (e.g., infrastructure, health care, and education) (Economist Intelligence, 2019; Giap et al., 2012). A city’s public goods are provided to citizens in a non-exclusive manner and without rivalry among citizens (The Global Economy, 2021).

In the attempt to improve the quality of life and expand the range of public goods and services within a city, technology is known to be an enabler with hundreds of initiatives aimed at introducing smart technologies in urban environments (Anthopoulos et al., 2016). Technologies can create digital platforms for accessing public goods and services that otherwise would remain unknown to citizens. Furthermore, public access to technologies themselves can contribute to an increased quality of life (e.g., free-of-charge Internet access) (Waters, 2016). Therefore, technology has a threefold role in improving citizens’ quality of life: by (a) enabling the creation of new technology-based public goods and services, (b) facilitating access to existing public goods and services, and (c) becoming part of the public goods and services portfolio offered to the citizens.

Putting technology centricity at the core of Smart City initiatives is known to have limitations in moving towards better cities (Hollands, 2008). Despite the technological advances within Smart City programs, citizens still face restrictions in accessing public goods and services. In response, research highlights the importance of moving beyond a technology focus to create citizen-centric solutions for future cities and their governance (D’Onofrio et al., 2019). However, multiple interpretations and manifestations of citizen centricity exist (e.g., Lee et al., 2014; Trencher, 2019; Yetis & Karakose, 2020), leading to a lack of conceptual clarity and guidance for organizations when implementing technology-based yet citizen-centric city initiatives (Curry et al., 2018; Azzam et al., 2019; Zhou et al., 2019).

In addition to technology centricity, an equally important barrier to taking stock of available technological advances is arguably provider centricity. The dominant issue in adopting Smart City solutions is a provider-centric understanding of the problem space which ignores how citizens engage with these solutions. Thus, a shift away from provider centricity is essential if the quality of life (in the city) is to be prioritized rather than seeing the service quality from a technological standpoint (Lee et al., 2014).

Any roadblocks to required city services compromise the citizen’s quality of life. Services may not be accessible due to high costs, inadequate location, and scarce availability at the time required. There is a demand to study how technologies could be used to overcome specific restrictions in the context of a city, as digital technologies have proven to be powerful solutions to overcoming restrictions in many parts of our broader life—e.g., elimination of cost to search (Google), time restrictions (e-government), or location (live streaming). Against this backdrop, this paper addresses the research question of *how the paradigm of technology-enabled elimination of restrictions in accessing city services can be conceptualized to enable the creation of new solutions.*

We answer the question by proposing a new conceptualization called City 5.0. We demonstrate its applicability and evaluate its usefulness for the analysis and development of technology-based solutions for accessing public goods and services. The paradigm of City 5.0 was first captured with the definition of a City 5.0 as “a liveable city that is (re)modelled with the aim of eliminating restrictions for its citizens by using digitalization for the provision of public goods and services” (Rosemann et al., 2021). However, City 5.0 has remained a rather vague concept, which has to date compromised its applicability. Therefore, we present a conceptualization of City 5.0 that focuses on capturing the interrelationships among its components in a framework. We then apply this framework to an example of citizen-centric and technology-based innovation in the public sector to demonstrate its applicability. Finally, we evaluate the framework’s usefulness through qualitative interviews with international members of the public sector.

## Positioning City 5.0 in smart city research

City 5.0 is a paradigm that emerged against the background of extensive research on the concept and shortfalls of smart cities (Rosemann et al., 2021). City 5.0 builds upon and enhances a citizen-centric perspective of smart cities and hence requires an understanding of the scope of extant smart city research.

There exists an ongoing discourse both in academia and practice about what a “smart city” actually entails (Chourabi et al., 2012; Hollands, 2008; Joss et al., 2019; Nilssen, 2019). This discourse is complicated by the fact that a smart city can be viewed as a *Soft City* (Sim, 2019), *Human Smart City* (Oliveira & Campolargo, 2015), or *Digital City* (Cocchia, 2014) depending on the chosen perspective. The initial introduction of the term in the 1990s had a strong technological focus on the applications of ICT in cities. Since then, in the course of progressing research (Dameri & Cocchia, 2013; Hosseini et al., 2018), two perspectives on smart cities have emerged: technology- and people-oriented perspectives (Brandt et al., 2018; Marrone & Hammerle, 2018).

The technology-oriented perspective refers to the use of ICT to support city activities and to provide the city with digital infrastructure. In this case, the focus is on enhancing prosperity, competitiveness, and effectiveness (Marrone & Hammerle, 2018). For example, according to Arrowsmith (2014), a smart city is a city that “has deployed or is currently piloting the integration of ICT solutions across three or more different functional areas of a city.” For Washburn and Sindhu, (2009, p. 2) a smart city implies “the use of smart computing technologies to make the critical infrastructure components and services of a city – which include city administration, education, healthcare, public safety, real estate, transportation, and utilities – more intelligent, interconnected, and efficient.” Smart computing technologies include software systems, server infrastructure, network infrastructure, and client devices (Washburn & Sindhu, 2009). Another central concept related to a smart city within this context is big data, in which the data assets, including real-time and near-real-time data streams from city infrastructures and sensor networks, enable the development of data-driven urban systems and services (Caird & Hallett, 2019).

By contrast, the people-oriented perspective put emphasis on human capital and prioritizing citizens’ needs (Marrone & Hammerle, 2018). The ultimate goal is to improve social, economic, and environmental sustainability, fostering inclusion, citizen participation, and liveability (Chourabi et al., 2012; Dameri & Cocchia, 2013; Hollands, 2008; Rosemann et al., 2021). In this respect, research has studied not only the role of ICT in smart cities but also the role of human capital/education, social and relational capital, and environmental awareness as crucial drivers of urban growth (Deakin, 2013). For instance, Komninos et al. (2015) argue that smart city applications should provide fresh and more effective ways of changing routines (daily activities of citizens, organizations, and governments).

Calls for more robust citizen-centric approaches have led to research that attempts to integrate the different perspectives on smart cities. Trencher (2019) argues that attention should be given to the emergence of what has been called the “Smart City 2.0,” a people-centric approach where smart technologies tackle social problems, address residents’ needs, and foster collaborative participation. Thus, the objectives of the technology and experiments with technologies should be to mitigate or solve social challenges, to enhance citizens’ well-being, and to improve public services. Anthopoulos et al. (2016) state that smart cities are intelligent digital ecosystems established in the urban space that are not limited to information and communication technology (ICT) and emphasize citizens’ involvement in the identification, proposal, and design of “smart solutions.”

More recently, the “4.0” suffix has been used to describe the integrated, contemporary use of digital systems in various contexts. Concepts such as Industry 4.0, Banking 4.0, or Health 4.0 (Lasi et al., 2014) describe smart environments which facilitate the low-cost production of a wide variety of personalized products and services.

We consciously chose the term City 5.0 to focus on the transition from 4.0 to 5.0. The notion of 5.0 is captured in prior research (Rosemann et al., 2021; Kowalkiewicz & Dootson, 2019) and expands the concept’s scope to include smart consumption and the dedicated use of technologies to address remaining constraints in the use of goods and services. In the context of a city, smart consumption looks more at how citizens can gain value from consuming public goods and services rather than focusing on production and implementation from the provider’s perspective. In this respect, City 5.0 directly connects to the notion of Industry 5.0 used by the European Commission (Breque et al., 2021, p.14), where “a human-centric approach [..] puts core human needs and interests at the heart of the production process. Rather than asking what we can do with new technology, we ask what the technology can do for us.” The idea of human centricity in Industry 5.0 fully aligns with the notion of City 5.0, wherein technology essentially has to be human-centric by resolving restrictions faced by citizens. Restrictions are inherently bound to humans (citizens) and represent issues relevant to them by capturing aspects that constrain citizens’ perception of liveability. As a paradigm, City 5.0 responds to this by implementing the idea of human centricity, which it seeks to ingrain through design as part of the resulting city solutions. Consistent with the European Commission’s notion of 5.0, a human-centric approach in cities puts core human needs and interests at the heart of city management. Rather than asking what we can do with new technology (in smart cities), we ask what the city can do for the citizens (eliminating restrictions).

With its focus on constraint-free liveability, the City 5.0 design paradigm connects to the understanding of well-being. The Organization for Economic Cooperation and Development (OECD) defines well-being as a result of local material conditions, quality of life, and sustainability (OECD, 2020). This implies the existence of initiatives to improve health, education, and social services, as well as enabling citizen participation, promoting positive environmental impacts, reducing vulnerability, and improving security. Striving for quality of life also requires improving work, housing, and infrastructure using information and communication technologies. However, Willis (2019) suggests that this focus on citizens has to date only resulted in a limited number of studies looking at how citizens are involved in and benefit from different smart city technologies.

Thus, City 5.0 is a step towards the “liveable” city—a concept related to the arguably less tangible concept of a “happy city” (Brdulak & Brdulak, 2017). City 5.0 does not contradict or neglect existing research on smart cities, but represents an additional lens as it extends the scope from a provider-centric view (how smart city technologies improve a city’s objectives—e.g., smart lights reduce electricity costs) to a citizen-centric view (how technologies overcome constraints for a citizen—e.g., suburban working hubs eliminate the need to enter a congested city). Similar to the notion of Six Sigma with its idealized state of quality to the extent that only 3.4 errors occur per one million opportunities, City 5.0 asks for an idealized city “free of constraints” for its citizens.

## Method

To answer our research question, we use a twofold approach. First, building a conceptualization requires assembling knowledge within a discourse universe, incorporating “all of the entities or elements that (a) are of interest and (b) may include (i) many entity worlds and (ii) entities that are not yet perceived or considered” (Weik, 2000). In other words, we need to identify the characteristics of this discourse universe, the entities in the universe, and the relationships among the entities. Therefore, we had to first understand the relevant concepts and then represent them in a comprehensive way. To this end, we conducted a series of workshops to gather knowledge from experts, where participants discussed their understanding and the perceived importance of constraints within city contexts. We then evaluated the conceptual model within real settings in which the discourse universe could be observed.

Workshops were conducted with a set of senior researchers from different academic fields (Appendix 1). All researchers had a long-standing record of projects and publications in information systems, business process management, and service science. They were selected on the basis of their expertise, recognition in academia and industry, and geographical diversity. They collectively brought together the necessary expertise to elicit, discuss, and reflect on the conceptualization. Co-authors of this work were among the workshop participants.

The strategy adopted was to start with discussions in small groups before then involving the whole team of experts. The first workshop was conducted with the participation of three experts (#1, #2, and #3 in the table in Appendix 1) during the International Conference on Information Systems (ICIS 2019). The goal was to identify and discuss the current restrictions in our individual projects that prevented citizens from consuming or co-creating a public service. The result was the derivation of the initial concepts.

The following workshops were conducted online at the beginning of 2020 with a combination of different participants in the group. The goal was to establish the different views on the issues related to public goods and services offered by governments and the relationships with technology and smart cities. The result was the jointly developed working draft of the City 5.0 design paradigm.

The concluding workshop counted on the participation of a bigger group (twelve participants from the table in Appendix 1). The goal was to consolidate the initial concepts, explicating and discussing different perspectives on restrictions in the form of a brainstorming session. This group then conceptualized restrictions on public services in more detail and identified strategies on how to manage them. The development was supported by input collected from twelve public administrations in the network (see Appendix 2). The goal was to determine the essence of a restriction, with participants proposing relevant components to model City 5.0. Afterwards, a discussion among the group members revealed whether these components would be helpful and have an appropriate level of abstraction for the model. The participants carefully kept the components exclusive and exhaustive.

We modeled the City 5.0 design paradigm with all its proposed elements and their relationships in a conceptual Entity Relationship Model (ERM) diagram following a constructivist approach. ERMs are used frequently as a means to model business environments (Scheer, 2013) for guiding and documenting conceptual work in interdisciplinary teams (Becker et al., 2010; Figl & Recker, 2016; Rosemann & Green, 2002). We then discussed the resulting model further and refined it repeatedly over 7 months until a consensus was achieved. The core of these activities was using the model as a boundary-spanning device to model and analyze particular restrictions found in our projects, leading us to instantiate, evaluate, and extend the model. Using this approach, we were able to add, rework, and eliminate concepts in our ERM until saturation was reached. Thus, while the concept was initially introduced based on the specific theoretical viewpoints of individuals, our consensus-oriented interpretivist research approach promoted the diversity of our viewpoints, which is an established epistemic theory of truth for conceptual modeling (Becker & Niehaves, 2007).

To evaluate the resulting conceptual model, we performed in the second step qualitative semi-structured interviews with members of the international public sector domain (Table 1) who have experience in using technology to create new public services or improve access to public goods. For the evaluation of our framework, we focused on the well-established concept of artifact usefulness (Prat et al., 2015). Specifically, we selected a qualitative approach as it represents the most common technique to evaluate usefulness (Prat et al., 2015). After being presented with the pre-written introduction to the conceptual framework of City 5.0, the interviewees answered a set of semi-structured questions regarding the framework’s usefulness that probed its benefits, challenges, and completeness. Subsequently, the interviews were transcribed, coded, and analyzed by the author team.

**Table 1:** List of the interviewees for the evaluation of the conceptual framework of City 5.0

Organization	Country	Interviewee position	Duration in minutes
City of Songdo (SDPMC) [SD]	South Korea	Representative Director and CEO	41
Spanish Ministry of Health [S]	Spain	Director of Healthcare Information Systems	74
IT Provider of the Municipality of São Paulo (PRODAM) [SP]	Brazil	Director of Innovation and Organizational Architecture	45
City of Muenster [M]	Germany	Head of Smart City Muenster	42
City of Paderborn [P]	Germany	Chief Digital Officer	45

## Conceptualization of City 5.0

Our vision of a city does not focus on technology applications as a means to an end. Instead, it sets the elimination of the citizens’ restrictions in accessing public goods and services as the aim. For an effective elimination of restrictions to take place, understandable, deployable, and assessable conceptualization of City 5.0 is required. A formalized conceptualization must entail constituents of the City 5.0 design paradigm and their relationships.

**Fig. 1:**
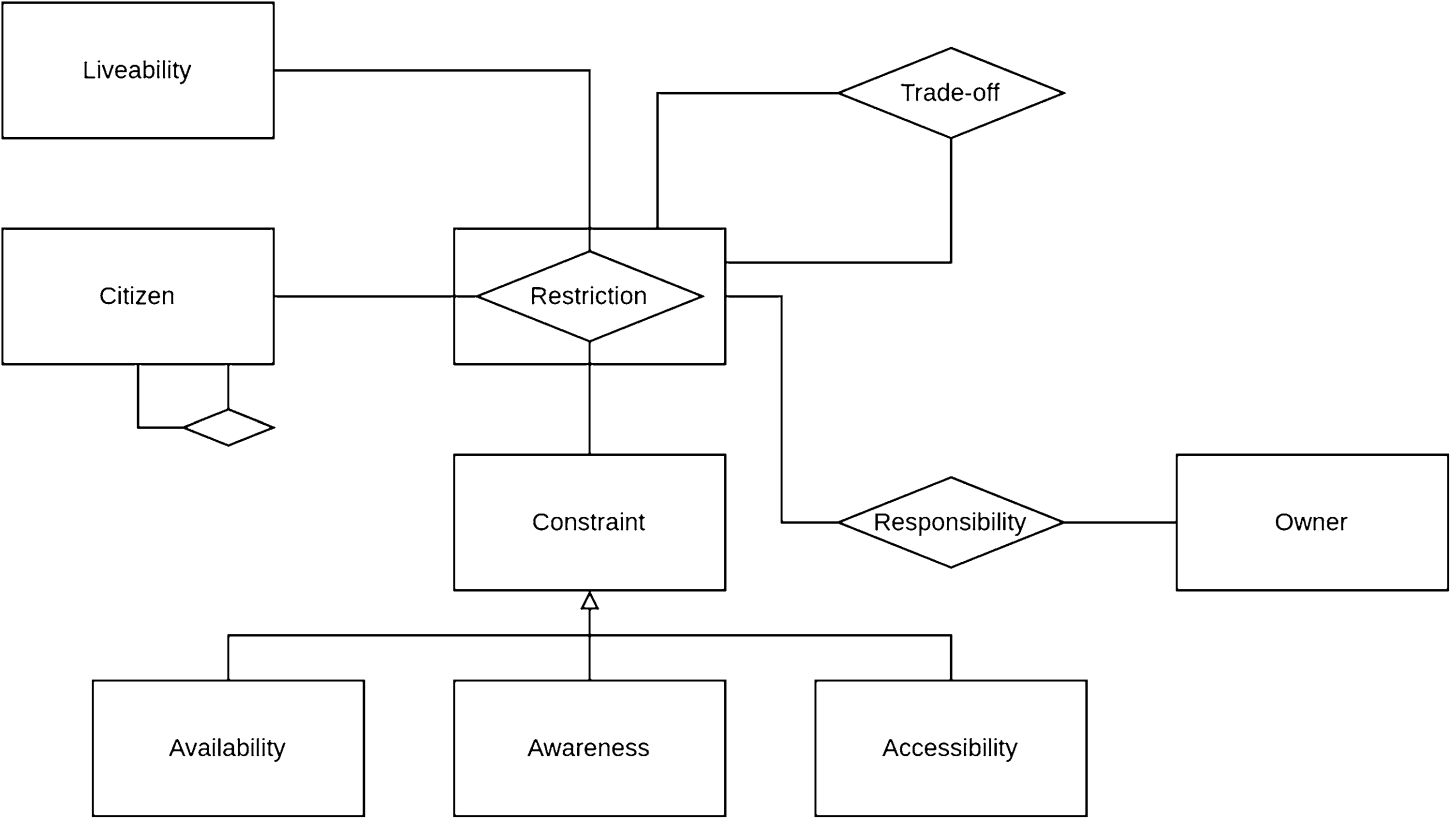
A fragment of the City 5.0 conceptual model

We chose a variation of the Entity Relationship Model (ERM) approach (Chen, 1976) as the modeling technique for our conceptual model. ERM allows for the semi-formal expression of relevant City 5.0 constituents (*entity types*) and their interrelation (*relationship types*) in a standardized way. Previous research used ERM for conceptualization, especially in the domain of ontology modeling (e.g., Rosemann & Green, 2002). Figure 1 illustrates a fragment of our model, which we use in the following to provide insights into the ERM notation and rationale. We then explain each of the components.

Entity types are drawn as rectangular boxes and represent collections of individual objects or notions (instances) that are categorized with the same representative class (e.g., citizen, liveability, owner, and constraint in the figure). Relationship types indicate associations between two or more entities and are graphically denoted as diamonds linked to entity types, the links being depicted by means of solid connecting lines. For example, distinct citizens can group and create networks (see the diamond connecting the citizen entity to itself). A restriction prevents a citizen group from achieving a desired quality of life or accessing public services. A restriction is thus a ternary relationship associating the citizen, constraint, and liveability entities, as we can see in Fig. 1. The three elements (liveability, citizen, constraint) constitute a restriction. Hence, a restriction is relevant and operationalizable if it is connected to a given liveability aspect lacking in a specific citizen group because of a specific constraint. Therefore, each individual restriction that has to be eliminated in the course of a City 5.0 initiative is a combination of the aforementioned entities.

During the peak of the COVID-19 pandemic, citizens affected by the disease faced the constraint of late access to therapies because of the high demand for medical services, thereby affecting perceived liveability in terms of health. Notice that the restriction diamond is surrounded by a rectangle. This additional visual element denotes a reinterpreted relationship type (also known as *associative entity*), meaning that the relationship can be reified (i.e., instantiated as a conceptual object per se) and have associations with other entities. In other words, the associative entity has a dual nature: it behaves as a relationship type and as an entity type. Note, moreover, that it is associated with an owner responsible for addressing it. Also, it is important to highlight that there can be a trade-off when it comes to restrictions (see the relation from and to the restriction entity in Fig. 1). For example, eliminating a restriction in connection to the safety of a citizen group by using citizen movement tracking can create a new restriction in connection to the infrastructure, discouraging citizens from entering tracked areas. Entity types can be specified on a more granular level via the *specialization/generalization* association, graphically depicted as a triangle. For example, a constraint collectively generalizes a concept that can be further specialized as an availability, awareness, or accessibility constraint, as depicted at the bottom of Fig. 1.

Note that some elements of ERM (e.g., attributes and cardinalities) are not used in our diagrams as they are required to represent fine-granular details and additional rules on the creation, update, and deletion of instances. Here, we focus solely on the conceptual representation of entities and their relationships (i.e., relevant concepts of the City 5.0 paradigm and the relationships between them, such as citizens facing constraints represented as two connected entity types—citizen and constraint—through a relationship type). See Appendix3 for more details on the adopted variant of ERM, we refer the reader to Appendix 3.

In the following, we first take a closer look at the constituting elements of the model—constraints, liveability, governance, and restriction management—to then develop our holistic model consisting of these elements.

### Constraints

**Fig. 2:**
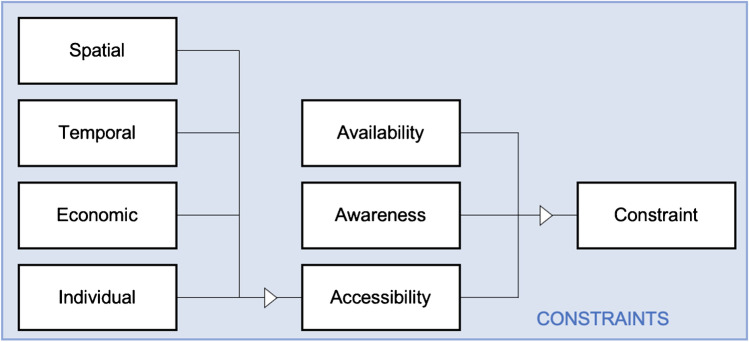
Constraint section of the City 5.0 conceptual model

*Constraints* are factors that can lower the performance of a system (Goldratt & Cox, 2016). In the context of a city system, citizens face restrictions when their liveability is impacted by a constraint. A *constraint* is an “abstract class” that only becomes relevant if it is experienced by a citizen group as a compromise of its perceived liveability. Access to public goods and services can be constrained for a group of citizens in three different ways: as *availability*, *awareness*, or *accessibility* constraint (see Fig. 2). The reason for separating constraints into three categories is that we aim to identify a mutually exclusive and collectively exhaustive representation of different types of constraints on a most abstract level, excluding those that do not fit one of the categories.

First, an existing public good or service can be known to the citizen group yet be *accessible* only in unreachable geographic areas (*spatial* constraint), at unsuitable times (*temporal* constraint), beyond the group’s purchasing power (*economic* constraint), or being subject to *individual* constraints (e.g., citizens being physically or cognitively impaired). Table 2 elaborates upon and provides examples of the respective constraint types following Rosemann et al. (2021). Addressing accessibility constraints thus implies striving towards the introduction of public goods for everyone, everywhere, at all times, and on any budget.

**Table 2:** Four availability constraints based on Rosemann et al. (2021)

Spatial accessibility constraints
Cities tend to grow rapidly, and lack of space creates bottlenecks. Related restrictions include long distances that must be traveled to work and competition for space (e.g., during peak hours). Long distances from homes to jobs are a physical restriction (Wachs & Kumagai, 1973), which is often explained with reference to the concept of “geography of opportunity” (Galster & Killen, 1995; Rosenbaum, 1995). Studies have shown that a geographical mismatch between a citizen’s home and job contributes to unemployment and creates dependence on public support programs (Opp, 2017; Osterman, 1991; Rosenbaum, 1995). Geographic information systems enable the examination of spatial characteristics in detail (e.g., through network analysis and accessibility mapping) and thus reveal novel perspectives on spatial constraints. Initiatives to overcome such constraints include distributed government offices that allow public servants to work at co-working spaces and “consume” public services closer to their homes/offices. Another example is the emergence of autonomous vehicles that will allow shorter distances between cars, leading to denser use of space
Temporal accessibility constraints
A city that is only “on” at certain times (e.g., public transport, shopping, government services) comes with temporal restrictions. These restrictions increasingly compromise liveability when global working models are used. The regulation of opening hours for retailers or governmental offices sets temporal restrictions that require new digital, self-serving solutions like the Amazon Go store and robotic public services
Economic accessibility constraints
Charges for consuming services in a city have an exclusive impact on citizens who cannot afford these services. For example, entering the City of London during peak hours is an expensive undertaking and an economic constraint for some citizens. The provision of free Wi-Fi services in public parks is an example of eliminating economic constraints, as is free public transportation in Tallinn (Estonia), and Luxembourg. Economic restrictions can inhibit innovation and business since barriers like poor or absent Internet connections can impede economic opportunities (Waters, 2016)
Individual accessibility constraints
A citizen’s physical and cognitive abilities can prevent him or her from using public goods and services. A physically impaired person can be restricted in reaching the location where he or she can consume a public good or service, and public services that require a certain level of cognitive ability can pose a barrier to cognitively impaired citizens. Digital technologies can help to eliminate these restrictions by introducing navigation aids for the physically impaired and specialized digital interfaces and digital assistants that lower the cognitive bar required for using public goods and services. It must be noted that lack of skill or knowledge in using digital technologies is part of individual accessibility constraints

Second, a public good or service can be *accessible,* yet a citizen group can lack *awareness* of its existence. Addressing the awareness constraints implies reversing the prevailing paradigm where citizens have to discover public goods and services. Instead, public goods and services have to “discover” the citizens and to make them aware of their existence. For instance, location-based security notifications and alerts of environmental hazards, bomb threats, or car traffic restrictions (e.g., permission to use only electric vehicles in selective city areas) are examples of services discovering the citizen and hence addressing the awareness constraints.

Third, the non-existence of a required public good or service represents an *availability* constraint. Examples would be the failures to provide trusted public data cloud to store data, freely accessible high-speed 5G networks, or public alert systems that notify citizens in times of crisis immediately. When facing this constraint, a required public good or service is not available to every citizen group. Addressing the availability constraint means introducing new public goods and services. Strikingly, the academia and practice have focused on this type of constraint looking for new services, yet often approach this challenge from the provider perspective with the aim of solving a pre-conceptualized citizens’ constraint.

### Liveability

**Fig. 3:**
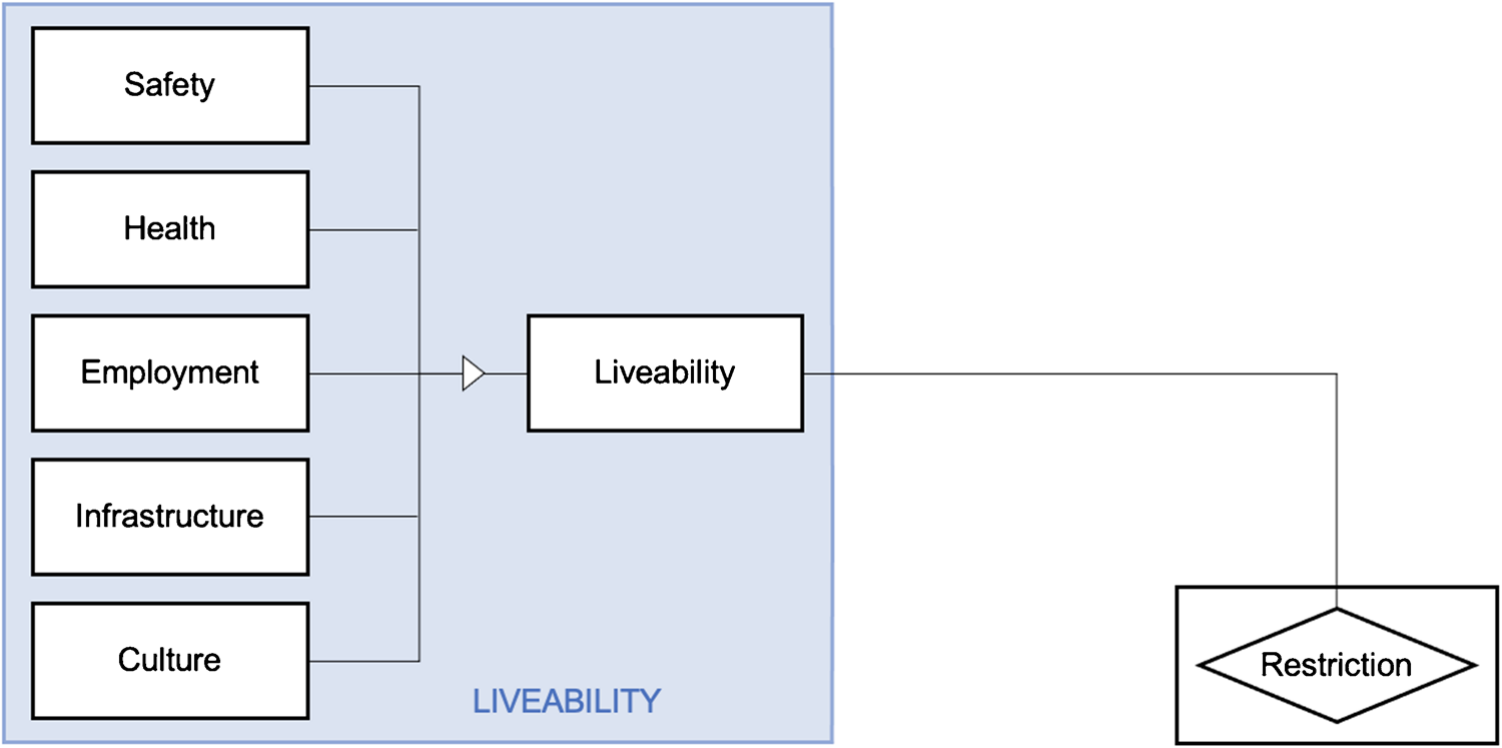
Liveability section of the City 5.0 conceptual model

*Liveability* is the degree to which a place is suitable or good for living in. A *constraint* that is experienced by a citizen group has to be related to a specific aspect of *liveability* (Fig. 3) in order to represent a *restriction* for a citizen group. In the following, we explain the rationale leading to our representation of liveability in the form of five distinct categories.

In academic and practical literature, the identification of which aspects determine the liveability in a city has been mainly undertaken in the context of city indicators, which are “quantitative, qualitative, or descriptive measures that enable information on a complex phenomenon, such as the dynamic urban environment, to be simplified into a form that is relatively easy to use and understand” (Huovila et al., 2019). Its use can be traced back to the early 90 s and the definition of a sustainability agenda called Agenda 21 at the UNCED meeting in Rio de Janeiro (Kitchin et al., 2015). The following decade saw the emergence of other quality-of-life indices, such as the Mercer’s annual quality of life survey, the Economist Intelligence Unit’s quality-of-life index, and the Global Liveable Cities Index. These indices aim at providing a ranking of cities based on the aggregation of several factors related to liveability, such as economic vibrancy, environmental friendliness, domestic security, socio-cultural conditions, and political governance (Giap et al., 2014). In parallel, the concept of smart city has given rise to additional categorizations that put more emphasis on the relevance of ICT and social participation in the context of a citizen’s quality of life. Some examples are the European Smart Cities Ranking (Giffinger et al., 2007) and the Bilbao Smart Cities Study (United Cities & Local Governments, 2012). Finally, several sets of city indicators have been published by such major international standardization bodies related to smart cities and sustainability as ISO 37120, ISO 37122, ITU-T 4901, 4902 and 4903, ETSI, or UN SDG 11 + (Huovila et al., 2019). The liveability aspects covered by these performance frameworks can be grouped into the five categories proposed in Rosemann et al. (2021): *stability*, *safety*, *and public governance*; *healthcare and social services*; *employment and economy*; *infrastructure, housing, and environment*; and *culture and education.* In our synthesis, we aim for a high-level aggregation to reduce the complexity of the model while allowing for all aspects of liveability to be mapped to one of the categories. Table 3 summarizes these categories and provides examples.

**Table 3:** Five liveability aspects based on Rosemann et al. (2021)

Stability, safety, and public governance (short: safety)
This category represents basic human needs and encompasses the prevalence of petty and violent crime, (traffic) accidents, the threat of terror, military conflict, and civil unrest/conflict. A shake-up of social harmony by conflict, e.g., can endanger stability. Here, public governance aims to provide effective policy and to act transparently and with accountability. A fair and efficient justice system fosters a society’s stability. Key Performance Indicators (KPIs), like the number of civil protection alarms, measure this dimension of liveability
Healthcare and social services (short: health)
Access to basic medical support (general practitioners, pharmacies) within a reasonable time and at a reasonable distance, along with advanced healthcare services like special medical support centers fosters citizens’ health. Social services, which refer to the social infrastructure communities need, comprise childcare, youth services, community centers, public toilets, outdoor public seating, and post offices. An example of a typical KPI is the availability and quality of healthcare services per capita
Employment and economy (short: employment)
The employment and economy dimension refers to open, business-friendly policies in the public domain, such as available space for new businesses and incentive schemes for entrepreneurs. Economic activities also depend on the local availability of viable, highly qualified employees. Employment and income are examples of KPIs, as they lead to growing or declining economic opportunities
Infrastructure, housing, and environment (short: infrastructure)
Infrastructure refers to a mixture of land use, which can include the following: transport networks, housing, and open spaces like playgrounds and public parks; road network, public (intermodal) transport, international transport, and travel connections; affordable, quality housing; reliable energy, water, and telecommunications, including high-speed Internet connectivity; and the environment, such as resilience to extreme weather conditions, green spaces, cleanliness of public areas, and low pollution. Examples of KPIs in this dimension are the average distance to well-connected transportation hubs and access to affordable housing that has access to all essential services
Culture and education (short: culture)
The culture and education dimension comprises sporting and cultural facilities and the availability of private and public education. Accessibility to and availability of educational opportunities refer not only to primary and secondary schooling, but also to tertiary education and learning centers for adults, including senior citizens. The provision of vibrant, cultural services catering for the demands of all facets of the local population is another indicator of cultural well-being. Exemplary KPIs to measure this category are access to affordable education, culture, and entertainment for the majority of the population

A relationship between a *citizen* group, a *constraint*, and a *liveability* aspect represents a restriction, which equates to a problem to be addressed through the collaboration of citizens, public administration, researchers, and private service providers. Permutations of constraints and liveability aspects define the scope of what City 5.0 initiatives can and should address. For instance, a City 5.0 initiative can focus on the identification of all four types of accessibility constraints (*economic*, *spatial*, *temporal*, and *individual*) that can influence the citizens’ situation with *infrastructure, housing*, and *environment*.

### Governance

**Fig. 4:**
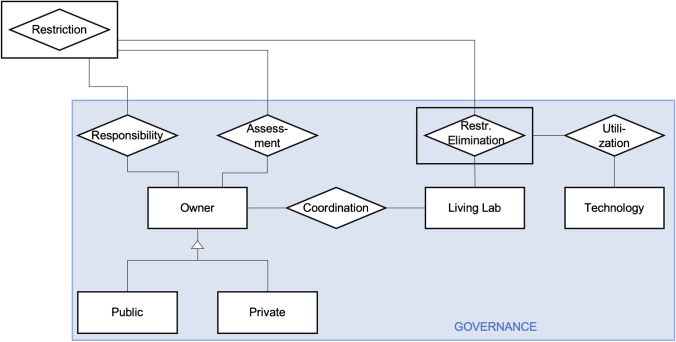
Governance section of the City 5.0 conceptual model

*Governance* addresses the structure and responsibilities of entities involved in eliminating citizens’ restrictions in accessing public goods and services. Restrictions have *owners* that can be either *public* or *private* entities. Owners have two roles. First, they *assess* the restrictions by identifying the restriction itself (which includes answering the questions “who is constrained?” “through which constraint?” and “which liveability aspect is constrained?”) and gauging the comparative impact of the restriction on *liveability*. Second, owners are *responsible* for addressing the restrictions and using the assessments to create priorities in restriction elimination initiatives. However, being responsible for the restrictions, owners do not necessarily *eliminate* or *resolve* restrictions on their own. Instead, the resolution can be performed by all kinds of dedicated collaborations. An abstraction from specific collaboration scenarios can be a *Living Lab.* The labs are the response to the complexity of the restrictions and the need for academic, professional, and public administration members cooperating with the citizens to eliminate a restriction. Actors participating in *Living Labs* are *coordinated* by restriction owners. Finally, technology can be used as a means to eliminate restrictions, likely in combination with other actions. This particular configuration of the governance-related concepts is primarily rooted in reflections upon current practice of public administrations, which often collaborate with industry. Such collaborations enable public administrations to compensate for the potential lack of skills and knowledge needed to create effective technology-based solutions. Figure 4 illustrates the *governance* aspects of the conceptual framework of City 5.0.

### Restriction management

**Fig. 5:**
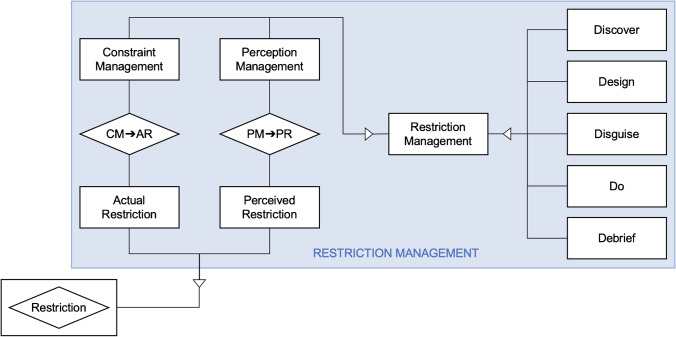
Restriction management section of the City 5.0 conceptual model

The nature of restrictions can vary as a combination of a constraint, citizen group, and liveability. As Fig. 5 illustrates, we discern restrictions as either *actual* (AR) or *perceived* (PR). We observe that whenever a public good or service is available and accessible yet a restriction is perceived, it does not mean that the restriction is irrelevant. On the contrary, such a restriction is concrete and relevant for the affected citizen group. For instance, safety measures adopted by public services might not counteract the perception that streets are unsafe if there have been isolated incidents so serious that they overshadow the average. Nevertheless, the way a perceived restriction is treated is different from the management of an actual restriction. For instance, a perceived restriction of being spatially constrained in accessing a good or service can in fact be an awareness constraint, and the good or service at hand can actually be accessible in the corresponding geographic area. To overcome such a restriction, introducing new public goods and services is unnecessary. Therefore, the *management of restrictions* requires the identification of the restriction’s nature in order to address the restriction through either *perception management* (PM) or *constraint management* (CM).

Perception and constraint management activities fall into one of the five phases of a restriction lifecycle management: *discover*, *design*, *disguise*, *do*, and *debrief*. Before a restriction can be eliminated, an in-depth understanding of the restriction is required. This understanding can be gained in the *discovery* phase. In this phase, a restriction can be analyzed in terms of whether it represents an actual or perceived restriction and diagnosed in terms of whether it is worth being resolved in light of the available resources and the restriction’s magnitude. Discovery includes a comparative analysis wherein a restriction is assigned a level of priority. If a restriction is selected as requiring a restriction elimination solution, the design of the solution follows. *Design* encompasses both a conceptual and a technical design. The focus is on creating IT artifacts capable of contributing to the elimination of the citizens’ restriction. In this step, digital technologies are identified that can be used to design the artifacts. Designed restriction elimination solutions can take two different shapes when they are deployed. First, a solution can focus on diverting citizens’ attention from the restriction. Such *disguise* tactics do not aim at “harming” or “misleading” the citizen or betraying their trust. Instead, they are effective when a citizen group can tolerate the actual restriction, and it is easier to design and deploy a solution that decreases the magnitude of the restriction’s perception. This phenomenon can be vividly demonstrated by the introduction of instruments that help decrease the perceived waiting time in queues or waiting rooms (e.g., for example, by having live art events or games at railway stations and bus stops). Although the actual temporal restriction of not being able to access a good or service during the waiting time remains the same, its perception can be influenced by enabling alternative activities. A variety of solutions using digital technologies can be applied this way to address non-existential restrictions. The alternative to disguise is to *do* something against the restriction directly. For instance, the introduction of new mobility services can eliminate a spatial restriction that prevents citizens from moving freely in a geographic area. Both perceived and actual restrictions can result in the decision to change the current landscape of public goods and services. The last part of the restriction management is *debriefing*. This raises awareness among citizens, highlighting when restrictions have already been (or are in the process of being) eliminated. Note that debriefing as a perception management approach can be used for addressing restrictions even without the application of active interventions within the restriction lifecycle management (e.g., designing, doing, and disguising). This can be the case when effective restriction elimination solutions are already in place and the restriction management can be reduced to the communication of the restriction elimination progress.

The restriction management lifecycle within City 5.0 might be perceived as counter-intuitive and can lead to untrivial strategies for addressing citizens’ restrictions, especially when it comes to addressing perceived restrictions. Thus, City 5.0 creates an opportunity space for non-provider-centric solutions, shifting attention away from a provider’s preconceptions regarding potential city solutions towards addressing relevant restrictions faced by citizens.

### Integrative conceptual model for City 5.0

**Fig. 6:**
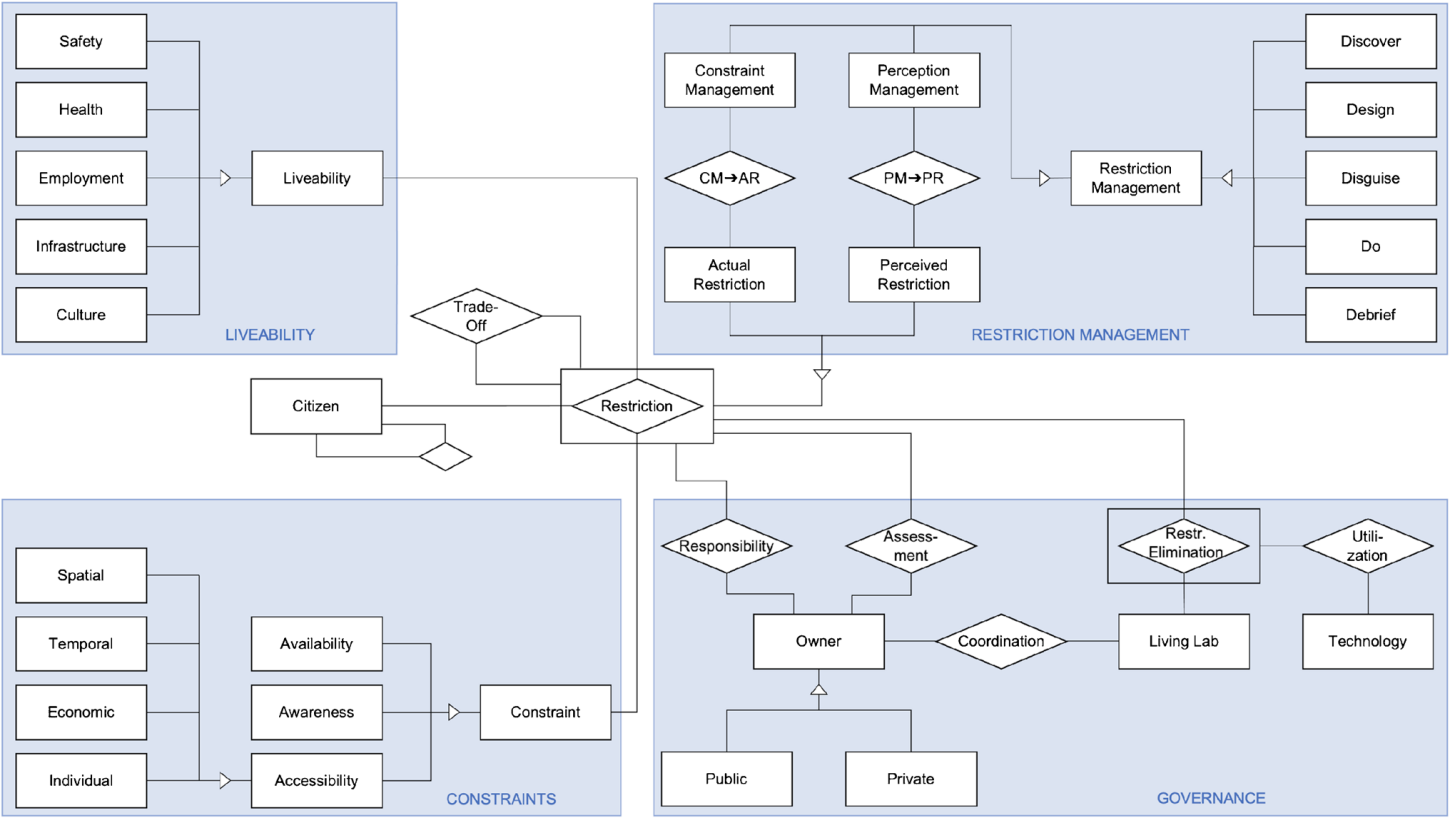
Conceptual representation of City 5.0 as an ERM diagram

Bringing all of these elements together, we develop our conceptual model as a combination of the concepts of *constraint*, *liveability*, *governance*, and *restriction management* to represent the City 5.0 design paradigm (Fig. 6). The four elements are connected through the entity type *restriction*.

Does this model contain all elements that are necessary to describe a restriction? Is it, in other words, exhaustive? As is the case for all conceptual models, such a question cannot be answered through automated verification, e.g., by an algorithm. We follow a consensus-oriented concept of correctness: we deem something appropriate when it stems from an agreement reached by the willing and knowledgeable (Kamlah and Lorenzen, p. 119). The “willing and knowledgeable” contributors are the group of authors who are all experts in their respective fields, and their findings are evaluated by the representatives of the cities involved. A model always has to be checked to see if it is adequate for the purpose of the study. Our purpose is to describe restrictions that may occur in a city and how these restrictions can be managed and overcome. As discussed above, the concept of liveability builds on an existing body of knowledge.

## Application of City 5.0

Applications of City 5.0 lead to restriction elimination solutions. Even though the conceptualization of the design paradigm has not yet been applied in dedicated projects, it can be observed in a plethora of pioneering city solutions that implicitly use digital technologies to eliminate restrictions. In the following, we will use an existing solution that embodies City 5.0—a telemedical service in Spain. We selected the case because it vividly demonstrates that the utility of using technologies in the context of public goods and services is not in the application of the technology as a goal in itself, but in the ability of the technology to help eliminate citizens’ restrictions arising from the changing context. The major data source for the case description is interviews with the project manager behind the service implementation.

### Servicio Andaluz de Salud—Case description

The Servicio Andaluz de Salud (SAS) is an organization that offers public healthcare services in Andalusia (Spain). This organization provides a set of citizen-healthcare services that enable patients to receive medical assistance and request information without having to leave their homes. These services are provided in the form of phone calls and web/mobile applications. Technology plays a central role. In response to the COVID-19 pandemic, for example, chatbots are used to provide general information and make a first pre-diagnostic assessment of the patient’s health condition. Technological support is used beyond mere information provision and enables communication and coordination with the health-service workers by managing appointments and even taking and transmitting images taken by the patients to describe their condition. In Granada, one of Andalusia’s largest cities, a total of 588,562 cases had been handled through this technological solution at the time of our study.

### Mapping the case to the conceptual model

The starting point for the case study of *Servicio Andaluz de Salud* (SAS) is the analysis of its *restrictions.* In this case, multiple restrictions are addressed that go back to the *health* aspect of liveability in combination with *accessibility* constraints. For instance, limitations on citizens during the COVID-19 pandemic represent a *spatial* constraint in accessing the physical locations of the healthcare centers. Therefore, a restriction is constituted by a combination of the general *citizen group*, the *health* aspect of liveability, and the *spatial* constraint. The physical inaccessibility is accompanied by a demand for healthcare services that is significantly higher than usual (by way of illustration, on the first day after confinement measures were introduced, the volume of calls to the emergency service center was ten times higher than usual). This creates a *temporal* constraint because many citizens may not have been able to access the service in these periods of high demand. Citizens infected with the virus faced a third restriction deriving from their *individual* inability to leave the house.

The *governance* of the restrictions in the case of SAS manifested itself in Andalusian Healthcare Service as a governmental institution taking the *ownership* of the restrictions. In this case, the restrictions were identified during the early days of the COVID-19 pandemic by SAS itself. The identification and *assessment* were based on the monitoring of the services that detected the unusually high demand of information and healthcare services by the citizens together with the new instructions given by the Spanish government imposing a lockdown. These circumstances triggered a collaboration between public administration, third sector providers (IT developers), and researchers in the form of a *Living Lab* which was *coordinated* by SAS. The third sector providers included both providers that were already working in technological solutions with SAS such as *Accenture plc* and others (such as IBM) that joined the team to collaborate on specific pandemic-related solutions. Different technological solutions were *utilized* in order to create *restriction elimination* solutions. These include a chatbot that helps respond to queries about COVID-19 (SAS, 2020a), employing an automatic call center to increase existing capacity and assist citizens with healthcare requests (SAS, 2020c), and a software tool that allows for the exchange of files between healthcare professionals and citizens (SAS, 2020b). The chatbot is a virtual assistant based on *IBM*’s AI technology *Watson* and can be accessed through the company’s public cloud. It answers questions from citizens relating to COVID-19, including symptoms, quarantine, and recommendations in case of infection. It includes mechanisms to protect the privacy of its users. Users can interact with the virtual assistant via the web or *Facebook*, *Telegram*, and *WhatsApp*, and is available around the clock, 7 days a week. The automatic call center (ACC), in turn, was introduced to support its existing human-operated counterpart. The ACC provided 60 new channels with virtual voice assistants and a capacity of 140,000 calls per week. It included Standard IVR, Intelligent IVR, and Virtual Voice Assistant functionalities based on *Accenture*’s AI facilities and the *Amazon* Transcribe-Polly speech-recognition synthesis. Finally, a new software tool called *Mercurio* was introduced to enable the secure file exchange between healthcare professionals and their patients. Both requests and files are received by citizens through their mobile phones via SMS. Citizens can use the same mechanism to send their files to the healthcare providers. The ACC is especially useful in areas such as dermatology, where the visualization of lesions is highly relevant for diagnosis and subsequent treatment. It was implemented by the IT department of SAS and integrated in *DIRAYA*, one of the main and biggest applications used in the SAS that integrates the electronic health records of more than seven million patients.

The *management* of restrictions in the case addresses *actual* restrictions. The *restriction management* lifecycle in the SAS example was focused on the *constraint management*. Specifically, restriction management began with *discovery*—identifying the specific nature of the restrictions to create effective and efficient solutions. The information for the discovery came both from affected citizens who voiced the challenges they faced and the SAS field work. As part of the discovery process, the restriction related to the need to reduce contact with other people and the general uncertainty surrounding the pandemic. Such a context increased information needs, thus driving the central value proposition for the citizens to be addressed in the course of restriction management. The subsequent *design* focused on the development of the aforementioned solutions—the chatbots and set of remote-working procedures. Here, the solutions were assessed in terms of their ability to eliminate the restrictions. The *disguise* mechanisms were not utilized because the discovery phase revealed that diverting citizens’ attention from the restriction is counter-productive when facing health-related issues that require a timely response. Instead, the *doing* in restriction management focused on promoting and distributing the developed restriction elimination solutions. The solutions provided citizens with the information they required concerning COVID-19 although this was generic rather than being personalized to each citizen. Furthermore, the solutions for remote diagnoses and attention helped to minimize contacts among patients and allowed them to access the service. After the deployment of the solution, the restriction management turned to *debriefing*—continuously communicating the progress of the restriction resolutions, for which the number of received enquiries (588,562 at the time of our study) and solved issues by means of remote diagnoses with *Mercurio* (40,000) served as proxies.

## Evaluation and discussion

The focus of the evaluation is twofold. First, it must support our framework’s usefulness for its primary target group in practice—public organizations. To evaluate our conceptual model, we focus on the established concept of usefulness (Prat et al., 2015) for the assessment of IS artifacts. We address three main topics: conceptual alignment with the target group’s understanding of the digital technology role in creating better cities, completeness, and specific benefits of the prospect framework application. Second, the evaluation must reveal potential misinterpretations of the framework and provide ideas for improving it. Since the most common forms of evaluating usefulness are qualitative techniques (Prat et al., 2015), we chose to conduct qualitative interviews. The presentation of the evaluation results will be followed by an examination of the main insights generated through the evaluation, as well as a discussion of the research contribution.

### Evaluation by experts in the public sector domain

“It is interesting to take [restrictions] as the starting point when thinking about technological developments because we often have […] available technology that we want to use no matter what. […] Instead of saying ‘Great, we have a technology, where can we use it?’ to ask which restrictions do our citizens have?”

[M:48]

The evaluation revealed that the interviewees recognized the main premises behind the City 5.0 design paradigm. They highlighted the still prevalent focus on providing technological solutions as a self-goal [M:48], spurious citizen orientation [S:180; P:29; SP:19], and the need to follow the example of the private sector where technologies are used to remove barriers in accessing goods and service in such sectors as retail (e.g., Amazon) and entertainment (e.g., Apple iTunes) [P:25].

However, despite being aware of these issues, the organizations interviewed for this study have not yet established a fitting paradigm in response to the observed situation around the use of digital technologies for creating public goods and services. Despite the recognition of the value behind the City 5.0 design paradigm (see the following evaluation of the benefits it brings), the explicit focus on citizens’ restriction is absent in the city initiatives of the interviewed partners. For example [P:5] reported that analyzing citizens’ restrictions “is not a perspective that we consider in our city.” A subsequent presentation of the City 5.0 design paradigm and the conceptual framework as a tool for establishing the elimination of citizens’ restrictions in designing future cities was met with approval from all interviewees [S:84; M:37; P:6; SD: 46; SP:19]. Specifically, interviewees explicitly highlighted the framework’s holistic nature and the ability to orchestrate policies [S:85], its strategic orientation [P:32], the important distinction in perceived and actual restrictions [P:39], the focus on public and private collaboration in Living Labs [S:270], and the framework’s completeness [S:87; SD:46; M:34].

The cornerstone of the framework’s appeal to the interviewees was its ability to map components to the elements of organizational conduct in the respective organizations and the terminology used to refer to these components. For instance, [S:84] commented that “while you were explaining the framework, I saw how it fits to what I observe in my area of responsibilities, and it all fits quite well.” [M:16] highlighted that the framework “fits my vocabulary and my professional domain.” The applicability to the real cases was outlined even in instances where not all the elements were important for a particular organization at this time: “some of the [framework elements] are absolutely present and others are ideal in a medium to long-term plan” [SP:19]. The interviewees were also asked about specific benefits to be expected if the City 5.0 design paradigm were implemented for eliminating citizens’ restrictions in the access of public goods and services by using digital technologies. They highlighted the seven benefits summarized in Table 4.

**Table 4:** Benefits in the context of usefulness of the City 5.0 application from the perspective of the interviewees

Benefit	Benefit description
Systematization of the complex process	Using the framework makes it possible to organize thoughts around a complex issue and allows you to execute it step by step
Fairness of problem treatment	Against the background of different topics that can be prioritized based on personal and organizational preferences, the systematic approaching of citizens’ restrictions enables fairness in the prioritization of issues to be addressed
Common view for people with different perspectives	Because heterogeneous stakeholders are involved in creating technology-based solutions for citizens, a common framework makes it possible to align the inputs and outputs of the joint efforts minimizing the effect of the actors’ heterogeneity
Helpful tool for understanding ineffectiveness of city solutions	The application of the conceptual framework in the context of already implemented technological solutions can provide insights into why citizens do not use them
Enforces acknowledgement of diverse citizen groups	Creation of technological solutions often comes with the implementing organization’s understanding of what the citizen requires. Explicit representation of various citizen groups aids recognition of faulty assumptions
Enforcement of citizen involvement and citizen centricity	The application of the conceptual framework implies citizen centricity because no solution can be created without identifying restrictions that will lead to specific citizen groups facing constraints in accessing public goods and services. These constraints are relevant to the perceived liveability of the affected citizen group
Complementarity	The City 5.0 paradigm has been described as an instrument that can be combined with the current approaches and methodologies used in the public sector to create public goods and services

The evaluation revealed the presence of three alternative understandings of “restrictions” in the vocabulary of the interviewed organizations. This finding opens up a conceptual space that was intentionally left out in the City 5.0 representation. Accordingly, instead of citizens’ restrictions in accessing public goods and services, public sector organizations tend to associate the term “restriction” with three aspects that deviate from the focus of City 5.0. First, public service providers can think about restrictions they face when creating their solutions (e.g., legal restrictions in the form of pressure to implement certain types of solutions, or lack of regulatory support [SP:8; M:36; S:6; P:12]). Second, the bureaucracy of the government system that hinders the efficient creation of public goods and services can be seen as a restriction in itself [SP:19]. Third, when thinking about public goods and services that are already available, public organizations can associate restrictions with attributes of the technological solutions that can hinder citizens’ access to these solutions [S:208] (for example, an excessively small font in the application offered by the government [S:127]). All these interpretations of restrictions shift the focus from actual restrictions citizens face in accessing public goods and services towards ones encountered by public service providers when creating new technology-enabled public goods and services. Following this shift of focus away from citizens towards organizations, a variety of internal restrictions were mentioned during the interviews. These include compliance with privacy laws [SD:44], contingency policies [SP:28], internal decision-making [M:36], and particularly the efficient leverage of city resources [SP:48]. The last point has been explicitly acknowledged as a problem because it can lead to taking the provider’s perspective, thus losing track of restrictions that the solutions should eliminate. Note that this viewpoint transition may not necessarily improve citizens’ quality of life [e.g., S:182].

## Discussion

The presence of three different types of restrictions identified through the evaluation of the conceptual framework (i.e., different understandings of what restrictions can represent aside from restrictions for a citizen in accessing public goods and services) is an important realization in the development of the City 5.0 design paradigm and the vision of an “ideal” city it implies. These different interpretations highlight the challenge for public organizations (which are tasked with creating digitally enabled solutions for citizens) to focus on restrictions that citizens face due to the lack of public goods and services, rather than on the restrictions that are associated with the organization itself or its outputs.

In fact, it appears that the public sector is dominated by the implicit idea that local administrations need to address their own restrictions first, then work on the restrictions they create in terms of citizens’ access to the technological solutions organizations provide, and finally turn their focus to the technology-independent restrictions citizen groups face because of insufficient or inadequate public goods and services. In light of this insight, the aim of City 5.0 can be seen in helping to reverse this order and set an explicit focus on citizens’ restrictions first. This focus shift, as highlighted during the evaluation, is likely to be enforced by the understanding of the restrictions as a combination of a citizen group and a constraint preventing the citizen group from improving one of the liveability aspects. Analyzing and addressing these restrictions has the potential to change the type of digitally enabled solutions that are created to remove the restrictions and to set the resolution of internal restrictions as a secondary objective. A good example of the result of such a shift would be a scenario where a local government improves the program for legally mandated child benefits (as in most European countries) by starting off with an analysis of the citizens’ restrictions, instead of focusing on the creation of a usable online application for childcare support and resolving internal restrictions that prevent them from offering this application in a timely manner. As a result, the elimination of a corresponding restriction according to the City 5.0 design paradigm will imply thinking about how automation and system integration (e.g., the integration of hospital systems and city-council systems) can be used to eliminate the need to access this online service. Thus, parents of a new-born would not be required to use technology to apply for child support, but would instead receive a letter advising them that they have already been automatically registered and that payments will arrive in due course.

To sum up, the conceptual representation of City 5.0 addresses all the central elements that together constitute restrictions. Its advantages are manifold. It can be used to identify restrictions by applying the constituents of the conceptual model as a checklist from different perspectives (for example, starting from the citizen group, the constraint, or the liveability aspect). Our City 5.0 model makes its constituents transparent and addresses them in a structured way. It thus allows for the identification of solutions to address citizens’ restrictions as a blueprint—an approach similar to a widely used business model canvas (Osterwalder & Pigneur, 2010). Moreover, the model can help to create innovative solutions around the restrictions faced by citizens or to reuse solutions for similar restrictions akin to the objective of design patterns (Gamma et al., 1993). We also note that making restrictions and potential solutions traceable facilitates business model innovations. Restrictions can often be observed in several other locations in a similar context, thereby serving as multiple opportunities for seamless adaptation and application scenarios—which can foster innovation as solutions often require a certain scale to be cost-efficient.

Our model’s inherently multi-perspective and transparent nature makes it beneficial for a wide range of stakeholders: public organizations and businesses alike can derive new service offerings to eliminate restrictions, politicians can frame problems they need to address in a broader scope, and citizens can make a more informed decision on the City 5.0 in which they want to live.

Our model is a conceptual artifact resulting from a design process with inherent degrees of freedom, based on decisions and contingency. Moreover, it is the result of a collaboration by the researchers who participated in the workshops. Since the participants are IS researchers, the ideas collected and structured in the model stem from common research areas. For example, the concept of “trade-off” is a term from risk management and the notion of “owner,” which has been integrated to take actions to actually resolve constraints, is similar to that of a risk owner. Thus, these views represent non-overlapping perspectives on the concepts. However, we acknowledge that participants from other backgrounds may have come up with other ideas and so produced a different-looking model.

Although the conceptualization yields numerous benefits, the City 5.0’s transferability into practice needs to be considered. There is a question of how existing smart city initiatives can benefit from the City 5.0 design paradigm. As already highlighted in the section dedicated to positioning City 5.0 within smart city research, City 5.0 does not require abandoning smart city-oriented thinking. Instead, it represents a design paradigm that frames the concept of smart city within a citizen-centric viewpoint—a perspective that goes beyond the capabilities of ICT to contribute to the development of future cities. This also leads us to the view that City 5.0 has the potential to bear immediate implications for existing smart city initiatives. By placing citizens’ restrictions in their ability to access public goods and services at the center of attention, existing solutions might need adjustments. It is likely that many existing smart city solutions lack the link to the relevant restrictions when put under scrutiny.

We observe that our study paves the way for a number of future endeavors. The retrospective analysis of successful initiatives in terms of their proximity to the City 5.0 paradigm and the evaluation of the framework by experts in the public sector are the foundation for future research. The need for initiatives that are explicitly based on City 5.0 calls for field studies and experiments to be conducted. Although the theoretical connection between the elimination of restrictions and citizens’ well-being is well grounded, observable improvements of liveability as a product of City 5.0-based initiatives will strengthen the power of the paradigm and facilitate its adoption. Academic, industrial, and governmental projects that use the City 5.0 lens to create new restriction elimination solutions will generate the data necessary for a subsequent analysis.

Furthermore, since the City 5.0 conceptualization considers both private and public bodies to be responsible for restrictions (and their elimination), the balance between them creates an important area of investigation. The issue emerges especially when restrictions that citizens face potentially fall into the responsibility area of private organizations. In such cases, private organizations can contribute to transforming commercial services into public services. In recent decades, we have already observed how private organizations can create public goods. For instance, Wikipedia represents a case where information—the lack of which leads to potential restrictions—is made a public good. Although underrepresented, such transformations of commercial goods and services into public ones through private organizations taking ownership of citizens’ restrictions represent a promising area of future research. This approach could potentially play an important role in easing the pressure on public finances caused by increasing the offering of goods and services.

Finally, we would like to point out an interesting phenomenon we observed during our research. In the course of our investigation, we realized that some restrictions could emerge when an existing public good or service starts to lose its characteristic as a public good or service by creating rivalry among citizens. For instance, in the case of *SAS*, the temporal restriction of not being able to access health services can be interpreted as a restriction emerging from the service demand itself, as it raises to the point at which it creates rivalry between citizens despite the non-exclusivity of the service. Dedicated studies are required to understand the ability of digital technologies to address the problem of public goods and services losing their public status through the emergence of rivalry.

## Conclusion

This article conceptualized and demonstrated applications of City 5.0, namely, the design paradigm of *a liveable city that is (re)modelled with the aim of eliminating restrictions for citizens by using digitalization for the provision of public goods and services*. The nexus of the proposed conceptualization is *restriction* as a combination of a constraint (e.g., accessibility of a particular service), a citizen group (e.g., a specific subset of citizens), and a specific liveability aspect that matters to the citizen group (e.g., safety). It illustrates how restrictions can be managed and how the governance of the restrictions is organized. City 5.0 represents a design paradigm required for achieving a wider involvement of citizens. It enforces the understanding of the restrictions in accessing public goods and services that citizens face before implementing any technology-enabled city solutions. The application of the City 5.0 design paradigm contributes to homogenizing the governance of future cities by aligning the efforts of public administrations, private organizations, and researchers around the aim of eliminating restrictions faced by citizens in accessing public goods and services. The work represents a contribution towards improving citizens’ quality of life and their inherent involvement in designing future cities.

## Appendix 1

Table 5

**Table 5:** List of researchers participated in the workshops

**#**	Name	Department	Research expertise (somehow related to smart city topics)	Iterations involved
1	Anonymized for the review process because of the senior researchers’ involvement as co-authors		Information Modeling Hybrid Value Creation Business Process Management E-Government Retail Information Systems	4
2	Anonymized for the review process because of the senior researchers’ involvement as co-authors		Knowledge Management Sustainability through Innovations in IS	4
3	Anonymized for the review process because of the senior researchers’ involvement as co-authors		Corporate innovation Revenue resilience	4
			Process management and trust management	
			Creation of compelling future worlds with today’s possibilities that make current practices obsolete	
4	Anonymized for the review process because of the senior researchers’ involvement as co-authors		Strategy and organizational design Digital innovation and transformation Management of collaboration in digital world	2
5	Anonymized for the review process because of the senior researchers’ involvement as co-authors		IT-supported services Business process management Design-oriented information systems	2
6	Anonymized for the review process because of the senior researchers’ involvement as co-authors		Conceptual modeling Knowledge graphs Open government	1
7	Anonymized for the review process because of the senior researchers’ involvement as co-authors		Service Science Smart service systems Business Process Management	3
8	Anonymized for the review process because of the senior researchers’ involvement as co-authors		Smart service systems Smart grid Geographic information systems Geospatial analysis	3
9	Anonymized for the review process because of the senior researchers’ involvement as co-authors		Business analytics Business information system Business process management Process mining Block chain Industrial AI	2
10	Anonymized for the review process because of the senior researchers’ involvement as co-authors		Process mining Digital twin healthcare process Smart city Urban computing	2
11	Anonymized for the review process because of the senior researchers’ involvement as co-authors		Process mining Declarative modeling Blockchain	2
12	Anonymized for the review process because of the senior researchers’ involvement as co-authors		Analysis and management of service level agreements Business process management Key performance indicators Cloud-based enterprise systems	3
13	Anonymized for the review process because of the senior researchers’ involvement as co-authors		Business Process Management Business Process Modelling, Monitoring and Analysis Process Performance Measurement Process Performance Indicators	3
14	Anonymized for the review process because of the senior researchers’ involvement as co-authors		Business Process Management Knowledge Management Collaborative systems Digital transformation	3

## Appendix 2

Table 6

**Table 6:** List of collaborating public administrations

**#**	Name	Country
1	Public Administration of the City of Vienna	Austria
2	Public Administration of the City of Nuremberg	Germany
3	Public Administration of the City of São Paulo	Brazil
4	Public Administration of the City of Paderborn	Germany
5	Public Administration of the City of Brisbane	Australia
6	Public Administration of the City of Muenster, Germany	Germany
7	Public Administration of the City of Pohang	South Korea
8	Public Administration of the City of Melbourne	Australia
9	Public Administration of the City of Latina	Italy
10	Public Administration of the City of Songdo	South Korea
11	Liechtenstein Center for Digital Capital Creation	Liechtenstein
12	Andalusian Health Service	Spain

## Appendix 3 ERM notation

### Purpose of conceptual ERM models

An Entity-Relationship Model (ERM) is a tool for representing a conceptual (data) model. A conceptual (data) model formally records relevant objects (*entities*) and *relationships* between them of the observed field on a conceptual level. Attributes can further specify the objects and relationships.

### Fundamental elements of an ERM

**Table:**

Element	Description
Entity	An informational representation of a real or artificial/abstract object (also: instance of an entity type), does not have a direct equivalent in the ERM
Entity type	Collection or *typing* of similar entities to a set (resp. class), illustrated by a *rectangle* in the ERM
Relation	Relationship between two entities; does not have a direct equivalent in the ERM
Relationship type	Typing of the relationship between two entity types at which one entity type can be related to itself (e.g., hierarchy), illustrated by a *diamond* in the ERM
Edge	Connects the entity types and relationship types; edges cannot be drawn between similar nodes (e.g., two entity types), illustrated by horizontal and vertical lines in the ERM
Attribute	Assigns typed characteristics to an entity type or a relationship type; attributes can be annotated by an elliptical node on an entity type or a relationship type
Cardinality	States how often an entity of an entity type A can be in a relation with an entity of an entity type B, if A and B are connected by a relationship type

### Subsumption or generalization/specialization

As we apply ERM for classifying certain aspects of restrictions, we make extensive use of subsumption to concretize these aspects. Our focus is not on describing data models to be implemented in databases but on facilitating classifications. We identified two types of relationships between granularities within entities that could be captured with the Enhanced Entity-Relationship (EER) Model depicted by Elmasri and Navathe (Fundamentals of Database Systems, 2011, Chapter 8). In the following figure, you can detect both notations: the left side depicts the notation we apply, and on the right side, we show the corresponding EERM diagram. Both models show that each *Restriction* is either classified as an *Actual* or *Perceived* restriction. There is total participation, i.e., each restriction can be classified this way.

If there are multiple relationships of this type, they all apply. For example, in the following figure with both notations, entity A is either of sub-type B or C, and either of sub-type D or E. In Fig. 6, an instance of *Restriction Management* could entail, e.g., *Perception Management* and *Discover*.

### Relationship types as entity types

Some relationships play a crucial role in our notation. Restrictions in particular show a dual nature of being an entity of their own and showing relations between entities. Hence, our notation resembles this by showing it as a relationship *and* an entity, e.g., *Restriction* in Fig. 1.

These relationship entities can be read as being an entity for edges that connect to relationships and as a relationship for edges that connect to entities. All other characteristics of the Chen notation and our extensions apply.

### Hierarchies

Hierarchies set entities into a superordinate/subordinate relationship. This produced a tree structure. An element can be subordinated to one element (of the same type) at most, but an element can be superordinate to an arbitrary number of elements of the same type.

## Footnotes

1 The reference format is a leading alphabetic code of the interview partner followed by a section of the transcribed interview.

## References

[CR1] Anthopoulos L, Janssen M, Weerakkody V (2016). A Unified Smart City Model (USCM) for smart city conceptualization and benchmarking. International Journal of Electronic Government Research.

[CR2] Arrowsmith, L. (2014). Smart cities: Business models, technologies, and existing projects. *Information Technology Services of IHS Technology*

[CR3] Azzam, A., Aryan, P. R., Cecconi, A., Di Ciccio, C., Ekaputra, F. J., Fernández, J. D., ... & Thurner, T. (2019). The CitySPIN Platform: A CPSS Environment for City-Wide Infrastructures. In *CPSS@ IOT* (pp. 57–64).

[CR4] Becker J, Niehaves B (2007). Epistemological perspectives on IS research: A framework for analysing and systematizing epistemological assumptions. Information Systems Journal.

[CR5] Becker J, Beverungen DF, Knackstedt R (2010). The challenge of conceptual modeling for product-service systems: Status-quo and perspectives for reference models and modeling languages. Information Systems and E-Business Management.

[CR6] Brandt T, Ketter W, Kolbe LM, Neumann D, Watson RT (2018). Smart cities and digitized urban management. Business and Information Systems Engineering.

[CR7] Brdulak, A., & Brdulak, H. (2017). *Happy city-how to plan and create the best livable area for the people*. Springer. 10.1007/978-3-319-49899-7

[CR8] Breque, M., De Nul, L., & Petridis, A. (2021). *Industry 5.0: Towards a Sustainable, Human-centric and resilient European industry*. Luxembourg, LU: European Commission, Directorate-General for Research and Innovation. https://ec.europa.eu/info/publications/industry-50_en. Accessed 15 Nov 2022.

[CR9] Caird SP, Hallett SH (2019). Towards evaluation design for smart city development. Journal of Urban Design.

[CR10] Chen PPS (1976). The entity-relationship model—Toward a unified view of data. ACM Transactions on Database Systems (TODS).

[CR11] Chourabi, H., Nam, T., Walker, S., Gil-Garcia, J. R., Mellouli, S., Nahon, K., Pardo, T. A., & Scholl, H. J. (2012). Understanding smart cities: An integrative framework. *2012 45th Hawaii International Conference on System Sciences* (pp. 2289–2297). IEEE

[CR12] Cocchia, A. (2014). Smart and digital city: A systematic literature review. In: Dameri, R., Rosenthal-Sabroux, C. (Eds.), *Smart city. Progress in IS* (pp. 13–43). Springer, Cham. 10.1007/978-3-319-06160-3_2

[CR13] Curry E, Hasan S, Kouroupetroglou C, Fabritius W, ul Hassan, U., & Derguech, W.  (2018). Internet of things enhanced user experience for smart water and energy management. IEEE Internet Computing.

[CR14] D’Onofrio, S., Habenstein, A., & Portmann, E. (2019). Ontological design for cognitive cities: The new principle for future urban management. In *Driving the Development, Management, and Sustainability of Cognitive Cities* (pp. 183–211). IGI Global. 10.4018/978-1-5225-8085-0.CH008

[CR15] Dameri, R. P., & Cocchia, A. (2013). Smart city and digital city: Twenty years of terminology evolution. *X Conference of the Italian Chapter of AIS (ITAIS 2013), 1*, 8.

[CR16] Deakin M (2013). Smart cities: Governing, modelling and analysing the transition - Google Books. Routledge.

[CR17] Economist Intelligence. (2019). *The global liveability index 2019*. https://www.eiu.com/n/the-global-liveability-index-2019/. Accessed 15 Nov 2022.

[CR18] Figl K, Recker J (2016). Process innovation as creative problem solving: An experimental study of textual descriptions and diagrams. Information and Management.

[CR19] Galster GC, Killen SP (1995). The geography of metropolitan opportunity: A reconnaissance and conceptual framework. Housing Policy Debate.

[CR20] Gamma E, Helm R, Johnson R, Vlissides J (1993). Design patterns: Abstraction and reuse of object-oriented design. Lecture Notes in Computer Science (Including Subseries Lecture Notes in Artificial Intelligence and Lecture Notes in Bioinformatics).

[CR21] Giap TK, Thye WW, Yam TK, Low L, Aw ELG (2012). Ranking the liveability of the world’s major cities: The global liveable cities index (GLCI). In Ranking the Liveability of the World’s Major Cities: The Global Liveable Cities Index (GLCI).

[CR22] Giap, T. K., Thye, W. W., & Aw, G. (2014). A new approach to measuring the liveability of cities: The global liveable cities index. In *World Review of Science, Technology and Sustainable Development* (pp. 176–196). 10.1504/WRSTSD.2014.065677

[CR23] Giffinger, R., Fertner, C., Kramar, H., & Meijers, E. (2007). Smart cities - Ranking of European medium-sized cities. In *Centre of Regional Science, Vienna UT*. https://www.smartcity-ranking.eu/download/city_ranking_final.pdf. Accessed 15 Nov 2022.

[CR24] Goldratt EM, Cox J (2016). The goal: A process of ongoing improvement.

[CR25] Hollands RG (2008). Will the real smart city please stand up? Intelligent, Progressive or Entrepreneurial?. City.

[CR26] Hosseini S, Frank L, Fridgen G, Heger S (2018). Do not forget about smart towns: How to bring customized digital innovation to rural areas. Business and Information Systems Engineering.

[CR27] Huovila A, Bosch P, Airaksinen M (2019). Comparative analysis of standardized indicators for smart sustainable cities: What indicators and standards to use and when?. Cities.

[CR28] Joss S, Sengers F, Schraven D, Caprotti F, Dayot Y (2019). The smart city as global discourse: Storylines and critical junctures across 27 cities. Journal of Urban Technology.

[CR29] Kamlah W, Lorenzen P, Robinson H (1984). Logical propaedeutic: Pre-school of reasonable discourse.

[CR30] Kitchin R, Lauriault TP, McArdle G (2015). Knowing and governing cities through urban indicators, city benchmarking and real-time dashboards. Regional Studies, Regional Science.

[CR31] Komninos N, Bratsas C, Kakderi C, Tsarchopoulos P (2015). Smart city ontologies: Improving the effectiveness of smart city applications. Journal of Smart Cities.

[CR32] Kowalkiewicz, M., and Dootson, P. 2019. Government 5.0: The future of public services. *QUT Chair in Digital Economy*. https://eprints.qut.edu.au/133743/. Accessed 15 Nov 2022.

[CR33] Lasi H, Fettke P, Kemper H-G, Feld T, Hoffmann M (2014). Industry 4.0. Business and Information Systems Engineering.

[CR34] Lee JH, Hancock MG, Hu MC (2014). Towards an effective framework for building smart cities: Lessons from Seoul and San Francisco. Technological Forecasting and Social Change.

[CR35] Marrone M, Hammerle M (2018). Smart cities: A review and analysis of stakeholders’ literature. Business and Information Systems Engineering.

[CR36] Nilssen M (2019). To the smart city and beyond? Developing a typology of smart urban innovation. Technological Forecasting and Social Change.

[CR37] Oliveira, Á., & Campolargo, M. (2015, January). From smart cities to human smart cities. *2015 48th Hawaii International Conference on System Sciences* (pp. 2336–2344). IEEE. 10.1109/HICSS.2015.281

[CR38] Opp SM (2017). The forgotten pillar: A definition for the measurement of social sustainability in American cities. Local Environment.

[CR39] Organisation for Economic Co-operation and Development (OECD). (2020).* Better Life Initiative: Measuring Well-Being and Progress*. https://www.oecd.org/sdd/OECD-Better-Life-Initiative.pdf. Accessed 15 Nov 2022.

[CR40] Osterman P (1991). Welfare participation in a full employment economy: The impact of neighborhood. Social Problems.

[CR41] Osterwalder A, Pigneur Y (2010). Business model generation: A handbook for visionaries, game changers, and challengers (Vol. 1).

[CR42] Portmann E, Tabacchi ME, Seising R, Habenstein A (2018). Designing cognitive cities. Springer International Publishing.

[CR43] Prat N, Comyn-Wattiau I, Akoka J (2015). A taxonomy of evaluation methods for information systems artifacts. Journal of Management Information Systems.

[CR44] Queensland Cabinet and Ministerial Directory (CMD). (2019). *Bolshoi ballet epic reaches across Queensland in free simulcast*. https://www.arts.qld.gov.au/news/bolshoi-ballet-epic-reaches-across-queensland-in-free-simulcast. Accessed 15 Nov 2022.

[CR45] Rosemann M, Green P (2002). Developing a meta model for the Bunge–Wand–Weber ontological constructs. Information Systems.

[CR46] Rosemann M, Becker J, Chasin F (2021). City 5.0. Business & Information Systems Engineering.

[CR47] Rosenbaum JE (1995). Changing the geography of opportunity by expanding residential choice: Lessons from the Gautreaux program. Housing Policy Debate.

[CR48] SAS. (2020a). Andalucía Crea un Asistente Virtual para Informar Sobre el Coronavirus, *Junta de Andalucía*. https://www.sspa.juntadeandalucia.es/servicioandaluzdesalud/todas-noticia/andalucia-crea-un-asistente-virtual-para-informar-sobre-el-coronavirus. Accessed 15 Nov 2022.

[CR49] SAS. (2020b). El SAS Impulsa la Consulta Telefónica para Pacientes de Atención Primaria, *Junta de Andalucía*. https://www.juntadeandalucia.es/presidencia/portavoz/salud/151320/AtencionPrimaria/SAS/ServicioAndaluzdeSalud/citas/consultas/coronavirus/Covid19. Accessed 15 Nov 2022.

[CR50] SAS. (2020c). Salud Responde Incrementa el Número de Horas de Operación ante el Aumento de la Demanda, *Junta de Andalucía.* https://www.juntadeandalucia.es/organismos/saludyfamilias/actualidad/noticias/detalle/242401.html. Accessed 15 Nov 2022.

[CR51] Scheer AW (2013). Architektur integrierter Informationssysteme: Grundlagen der Unternehmensmodellierung. Springer-Verlag.

[CR52] Sim D (2019). Soft city: Building density for everyday life.

[CR53] The Global Economy. (2021). *Public Services Index - Country Rankings.* https://www.theglobaleconomy.com/rankings/public_services_index. Accessed 15 Nov 2022.

[CR54] Trencher G (2019). Towards the smart city 2.0: Empirical evidence of using smartness as a tool for tackling social challenges. Technological Forecasting and Social Change.

[CR55] United Cities and Local Governments. (2012). *Smart cities study: International study on the situation of ICT, innovation and knowledge in cities*. https://issuu.com/uclgcglu/docs/smart_cities_2017_en. Accessed 15 Nov 2022.

[CR56] United Nations. (2018). *68% of the world population projected to live in urban areas by 2050, says UN*. https://www.un.org/development/desa/en/news/population/2018-revision-of-world-urbanization-prospects.html. Accessed 15 Nov 2022.

[CR57] Wachs M, Kumagai TG (1973). Physical accessibility as a social indicator. Socio-Economic Planning Sciences.

[CR58] Washburn D, Sindhu U, Balaouras S, Dines RA, Hayes N, Nelson LE (2009). Helping CIOs understand “smart city” initiatives. Growth.

[CR59] Waters, J. (2016). Accessible cities: From urban density to multidimensional accessibility. In *Rethinking Sustainable Cities: Accessible, Green and Fair* (1st ed., pp. 11–60). Bristol University Press. 10.51952/9781447332855.ch002

[CR60] Weik, M.H. (2000). Discourse universe. In: *Computer Science and Communications Dictionary*. Springer, Boston, MA. 10.1007/1-4020-0613-6_5215.

[CR61] Willis KS (2019). Whose right to the smart city?.

[CR62] Yetis, H., & Karakose, M. (2020). A cyber-physical-social system based method for smart citizens in smart cities. *2020 24th International Conference on Information Technology (IT)* (pp. 1–4). IEEE. 10.1109/IT48810.2020.9070685

[CR63] Zhou Y, Yu FR, Chen J, Kuo Y (2019). Cyber-physical-social systems: A state-of-the-art survey, challenges and opportunities. IEEE Communications Surveys & Tutorials.

